# The DNA mismatch repair gene hMSH2 is a potent coactivator of oestrogen receptor *α*

**DOI:** 10.1038/sj.bjc.6602614

**Published:** 2005-05-10

**Authors:** O Wada-Hiraike, T Yano, T Nei, Y Matsumoto, K Nagasaka, S Takizawa, H Oishi, T Arimoto, S Nakagawa, T Yasugi, S Kato, Y Taketani

**Affiliations:** 1 Department of Obstetrics and Gynecology, Graduate School of Medicine, The University of Tokyo, Hongo 7-3-1 Bunkyo-ku, Tokyo 113-8655, Japan; 2 SORST, Japan Science and Technology, Honcho 4-1-8, Kawaguchi, Saitama 332-0012, Japan; 3 Institute of Molecular and Cellular Biosciences, The University of Tokyo, Yayoi 1-1-1 Bunkyo-ku, Tokyo 113-0034, Japan

**Keywords:** hMSH2, HNPCC, ER *α*, ER *β*, coactivator, transactivation

## Abstract

The DNA mismatch repair gene is a key regulator in the elimination of base–base mismatches and insertion/deletion loops (IDLs). Human MutS homologue 2 (hMSH2), originally identified as a human homologue of the bacterial MutS, is a tumour suppressor gene frequently mutated in hereditary nonpolyposis colorectal cancer. Hereditary nonpolyposis colorectal cancer is characterised by the early onset of colorectal cancer and the development of extracolonic cancers such as endometrial, ovarian, and urological cancers. Oestrogen receptor (ER) *α* and *β* are members of a nuclear receptor (NR) superfamily. Ligand-dependent transcription of ER is regulated by the p160 steroid receptor coactivator family, the thyroid hormone receptor-associated proteins/the vitamin D receptor-interacting proteins (TRAP/DRIP) mediator complex, and the TATA box-binding protein (TBP)-free TBP associated factor complex (TFTC) type histone acetyltransferase complex. Here, we report the interaction between ER *α*/*β* and hMSH2. Immunoprecipitation and glutathione-*S*-transferase pulldown assay revealed that ER *α* and hMSH2 interacted in a ligand-dependent manner, whereas ER *β* and hMSH2 interacted in a ligand-independent manner. Oestrogen receptor *α*/*β* bound to hMSH2 through the hMSH3/hMSH6 interaction domain of hMSH2. In a transient expression assay, hMSH2 potentiated the transactivation function of liganded ER *α*, but not that of ER *β*. These results suggest that hMSH2 may play an important role as a putative coactivator in ER *α* dependent gene expression.

The mismatch repair (MMR) system was originally identified in bacteria, and its inactivation results in an increase in the rate of spontaneous mutations owing to the inability to repair the replication errors. The best understood function of the MMR system is to eliminate single-base mismatches and insertion–deletion loops (IDLs) that may arise during DNA replication by DNA polymerases (Glickman and Radman, 1980; Modrich, 1997). In the first step of the repair process in humans, DNA mismatches are efficiently recognised by protein heterodimers containing human MutS homologue 2 (hMSH2). The most abundant complex is hMutS*α*, a heterodimer containing hMSH2 and hMSH6. The quantitatively more minor recognition factor hMutS*β* is a heterodimer comprising hMSH2 and hMSH3. The subsequent step of repair involves hMutL*α*, a heterodimer comprising human MutL homologue 1 (hMLH1) and hPMS2, which interacts with hMutS*α*, and directs the removal and replacement of a long stretch of DNA containing the mismatches or IDLs (Ban *et al*, 1999). The functions of hMSH3 and hMSH6 are thought to be not critical for the repair of DNA mismatches because the disruption of either hMSH3 or hMSH6 leads to a mild mutator phenotype (Marsischky *et al*, 1996). In contrast, hMSH2 is indispensable to the recognition of mismatches and the inactivation of hMSH2 is responsible for a phenotype referred to as replication error or microsatellite instability (Bubb *et al*, 1996).

Hereditary nonpolyposis colorectal cancer (HNPCC) acounts for approximately 2–5% of all colorectal cancer (Mitchell *et al*, 2001), and remains the most common of the recognised inherited colorectal cancer syndrome. Hereditary nonpolyposis colorectal cancer is predisposed to the early onset of colorectal cancer and the development of extracolonic neoplasms including endometrial, ovarian, urological, and upper gastrointestinal cancers (Rodriguez-Bigas *et al*, 1996). The identification of MMR gene mutations associated with HNPCC revealed a novel mechanism of cancer pathogenesis (Fishel *et al*, 1993; Leach *et al*, 1993). Hereditary nonpolyposis colorectal cancer is associated with germ-line mutations of the MMR genes, most commonly hMSH2 and hMLH1. The hMSH2 gene, located on chromosome 2p22–p2, consists of 15 exons and encodes a relatively large protein of 934 amino acids. The mutations of hMSH2 represent approximately 40% of the mutations detected in HNPCC kindreds. Among the 154 different hMSH2 mutations that have been analysed approximately 80% of the hMSH2 mutations lead to premature termination of the hMSH2 gene product. The truncated hMSH2 gene product may lose its critical functions (Drotschmann *et al*, 1999), which is related to the development of HNPCC.

Oestrogen receptor is a member of the nuclear hormone receptor gene superfamily and acts as a ligand-induced transcription factor (Mangelsdorf *et al*, 1995). Oestrogen receptor is stimulated by two distinct activation regions, activation function-1 (AF-1) and AF-2. Activation function-1, which is located in the N-terminal A/B domain, is constitutively activated in cell-type and promoter-specific manner (Metzger *et al*, 1995). Activation function-2 is located in the C-terminal ligand-binding domain and exerts a ligand-dependent transcriptional activation. Activation function-1 and AF-2 activate transcription independently and act synergistically (Lopez *et al*, 1999). Oestrogen receptor *α* contact the general transcription factors either directly or indirectly via coactivator proteins, including AF-1 coactivator p300 (Kobayashi *et al*, 2000) and p68/p72 (DEAD box protein) (Watanabe *et al*, 2001). The ligand-dependent activation of ER *α* requires ligand-dependent association of AF-2 coactivator complex, including p300/CBP, pCAF, the p160 family members SRC-1/TIF-2/pCIP/AIB-1/GRIP1/ACTR (McKenna *et al*, 1999), the thyroid hormone receptor-associated proteins/the vitamin D receptor-interacting proteins (TRAP/DRIP) (Rachez *et al*, 2000; Kang *et al*, 2002), the transformation/transcription domain-associated protein (TRRAP), and the histone acetyltransferase GCN5 (Yanagisawa *et al*, 2002).

We have recently revealed that breast cancer susceptibility gene 1 (BRCA1), hMSH2, and GCN5 formed a complex to work on DNA repair (unpublished data). Current studies have implicated DNA repair proteins, including the T:G mismatch specific thymine DNA glycosylase (Chen *et al*, 2003), and the xeroderma pigmentosum complementary group D (XPD) (Keriel *et al*, 2002) in the transcription regulation by nuclear receptors (NRs). On the basis of the report showing the transcriptional squelching between BRCA1 and ER *α* (Fan *et al*, 1999), we investigated the functional interaction between hMSH2 and ER *α*.

## MATERIALS AND METHODS

### Cell culture

Oestrogen receptor *α*/*β*-positive T-47D (HTB-133) and ER *β*-positive MDA-MB-231 (HTB-26) human breast cancer cell lines were purchased from the American Type Culture Collection (ATCC, Manassas, VA, USA). Oestrogen receptor *α*-positive Ishikawa human endometrial cancer cell line was kindly provided by Dr M Nishida (Tsukuba University, Tsukuba, Japan), and hMSH2-deficient HEC59 human endometrial cancer cell line was a generous gift from Dr H Kuramoto (Kitasato University, Sagamihara, Japan). These cells were maintained in Dulbecco's modified Eagle's medium (DMEM) (Sigma Aldrich, St Louis, MO, USA) supplemented with 10% fetal bovine serum (Sigma Aldrich).

### Immunoprecipitation

Formation of ER *α*–hMSH2 complex in Ishikawa cells and ER *β*–hMSH2, BRCA1–hMSH2 (Wang *et al*, 2001) complex in MDA-MB-231cells were analysed by co-immunoprecipitation using the specific antibody raised against human ER *α* (G-20), human ER *β* (H-150), and human BRCA1 (C-20), followed by immunoblotting using the anti-hMSH2 antibody (N-20). All the antibodies were purchased from Santa Cruz Biotechnology (Santa Cruz, CA, USA). The 3 × 10^6^ cells were suspended in 1000 *μ*l of TNE buffer (10 mM Tris-HCl (pH 7.8), 1% Nonidet-P40, 0.15 M NaCl, 1 mM EDTA, 1 *μ*M phenylmethylsulphonyl fluoride, and 1 *μ*g ml^−1^ aprotinin). The cells were disrupted by repeated aspiration through 27-gauge needle and cellular debris were precipitated by centrifugation at 12 000 r.p.m. for 30 min at 4°C. The whole supernatant was applied for immunoprecipitation with 3 *μ*g of the antibodies to ER *α*, ER *β*, BRCA1, or IgG. Then, 30 *μ*l of protein G sepharose™ 4 Fast Flow (Amersham Biosciences, Buckinghamshire, UK) was added, and subsequently the bound proteins were purified as a protein complex. After washing with TNE buffer four times, the interacting proteins were separated by 7.5% SDS–polyacrylamide gel electrophoresis, transferred onto Hybond™-ECL™ nitrocellulose membrane (Amersham Biosciences), and detected by Western blotting with the anti-hMSH2 antibody. ECL detection was performed according to the manufacturer's recommendations.

### Plasmid construction

TRAP220 and SRC-1 expression vectors were described previously (Fujita *et al*, 2003; Wada *et al*, 2004). Human MutS homologue 2 expression vector was constructed from human testis library (Clontech, Palo Alto, CA, USA). Fragments of hMSH2 were inserted into pcDNA FLAG vector derived from pcDNA 3 (Invitrogen, Carlsbad, CA, USA). Human ER*α* AF-1 (1–180), ER *α* AF-2 (263–595), ER *β* AF-1 (1–149), and ER *β* AF-2 (248–530) vectors were described previously (Fujita *et al*, 2003). Reporter constructs (17M8 *β* globin-luc) have been described previously (Wada *et al*, 2004).

### Glutathione-*S*-transferase-pulldown assay

Glutathione-*S*-transferase (GST) fusion proteins or GST alone were expressed in *Escherichia coli* and bound to glutathione-sepharose 4B beads (Amersham Biosciences). Immobilised GST-ER *α*/*β* AF-1 or AF-2 fusion proteins were preincubated for 30 min in NET buffer (20 mM Tris-HCl pH 7.5, 200 mM NaCl, 1 mM EDTA) with or without 17-*β* estradiol (10−6 M). The GST proteins were incubated at 4°C with [^35^S]methionine (Amersham Biosciences)-labeled proteins expressed by *in vitro* translation using the TNT-coupled transcription–translation system (Promega Co., Madison, WI, USA). After 1 h incubation, unbound proteins were removed by washing the beads three times in NET buffer with 0.5% Nonidet P-40 and 1 mM phenylmethylsulphonyl fluoride. Specifically bound proteins were eluted by boiling in SDS sample buffer and analysed by 7.5–15% SDS–polyacrylamide gel electrophoresis and autoradiography. Polyacrylamide gels were stained briefly with Coomassie Brilliant Blue to verify the loading of equal amounts of fusion proteins prior to drying and autoradiography.

### Luciferase assay

At 2 days before transfection, the medium was changed to phenol red-free DMEM containing 5% charcoal stripped fetal bovine serum. Transfection was performed with PolyFect reagent (Quiagen, Inc., Chartsworth, CA, USA) according to the manufacturer's recommendations. For luciferase assay, 250 ng 17M8 *β-*globin-luc plasmid was cotransfected with 250 ng pM ER *α* AF-2 or pM ER *β* AF-2. Indicated expression vectors were also cotransfected with ER vectors. As an internal control to equalise transfection efficiency, 5 ng pRL CMV vector (Promega Co., Madison, WI, USA) was cotransfected in all the experiments. Individual transfections, each consisting of triplicate wells, were repeated at least three times (Wada *et al*, 2004).

## RESULTS

### ER *α*/*β* and hMSH2 interacts *in vivo*

**Figure 1:**
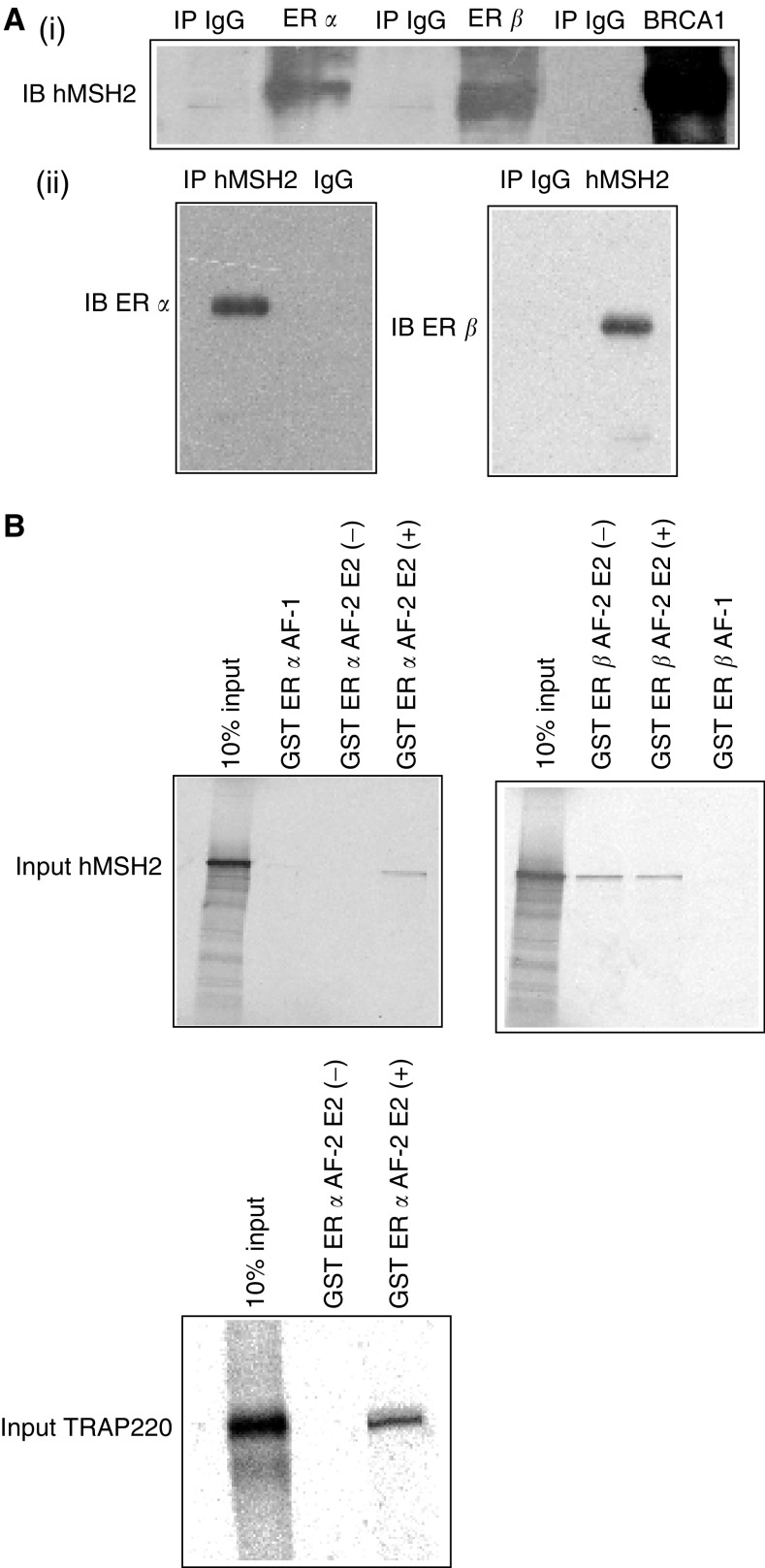
*In vivo* association between hMSH2 and ER *α*/*β* and *in vitro* association between hMSH2 and ER *α*/*β.* (**A**) (i) Ishikawa cells were lysed and subjected to immunoprecipitation (IP) with the antbodies to ER *α* and IgG. MDA-MB-231 cell lysate was immunoprecipitated with the antbodies to ER*β* BRCA1 and IgG. The immunoprecipitates were separated by SDS–PAGE and analysed by immunoblotting (IB) with the anti-hMSH2 antibody. (ii) Reciprocal IP was performed to detect endogenous hMSH2-ER *α* and hMSH2-ER *β* interactions by IB. (**B**) *In vitro* translated ^35^S-labelled hMSH2 was pulled down by GST-ER *α*/*β* AF-1 or GST-ER *α*/*β* AF-2. At the same time, *in vitro* translated TRAP220 was incubated with GST-ER *α* AF-2. The mixtures were washed and subjected to SDS–PAGE and analysed.

To determine whether endogenous hMSH2 protein interacted with ER *α*, ER *β*, and BRCA1 in the cultured human cells, we performed immunoprecipitation assays by using the antibodies raised against ER *α*, ER *β*, and BRCA1. Oestrogen receptor *α* was immunoprecipitated in Ishikawa cell lysate. Oestrogen receptor *β* and BRCA1 were immunoprecipitated in MDA-MB-231 cell lysate. Complex formation of the precipitated proteins was confirmed by Western blotting. Immunoblotting revealed the exsistence of hMSH2 in the cell lysate immunoprecipitates (Figure 1A(i)), which supports our hypothesis that hMSH2 physically associates with ER *α* and ER *β* in living cells. These results were further confirmed by reciprocal immunoprecipitation with the specific antibody raised against hMSH2. Immunoblotting again revealed the exsistence of ER *α* and ER *β* (Figure 1A(ii)).

### ER *α* and hMSH2 interact *in vitro* in a ligand-dependent manner, and ER *β* and hMSH2 interact *in vitro* in a ligand-independent manner

To address the functional importance of the hMSH2-ER *α* and hMSH2-ER *β* interaction, *in vitro* translated hMSH2 in the presence of [^35^S] methionine was incubated with GST-fused ER *α*/*β* AF-1 or ER *α*/*β* AF-2. As clearly shown in Figure 1B, the liganded GST-fused ER *α* AF-2 protein possessed the ability to retain hMSH2 on the column. On the other hand, GST-fused ER *α* AF-2 protein without the ligand, and GST-fused ER *α* AF-1 protein lacked the ability to retain hMSH2 on the column. Liganded ER *α* AF-2 possessed the ability to retain TRAP220, one of the coactivator of ER *α*. These data indicated that hMSH2 directly interacts with ER *α* AF-2 in a ligand-dependent manner, similar to TRAP220. Glutathione-*S*-transferase-fused ER *β* AF-2 protein possessed the ability to retain hMSH2 on the column regardless of the presence of ligand (Figure 1B). Glutathione-*S*-transferase-fused ER *β* AF-1 protein lacked the ability to retain hMSH2 on the column. These results indicated that hMSH2 directly interacts with ER *β* AF-2 in a ligand-independent manner.

### Mapping of the region of hMSH2 that interacts with ER *α* AF-2 and ER *β* AF-2

**Figure 2:**
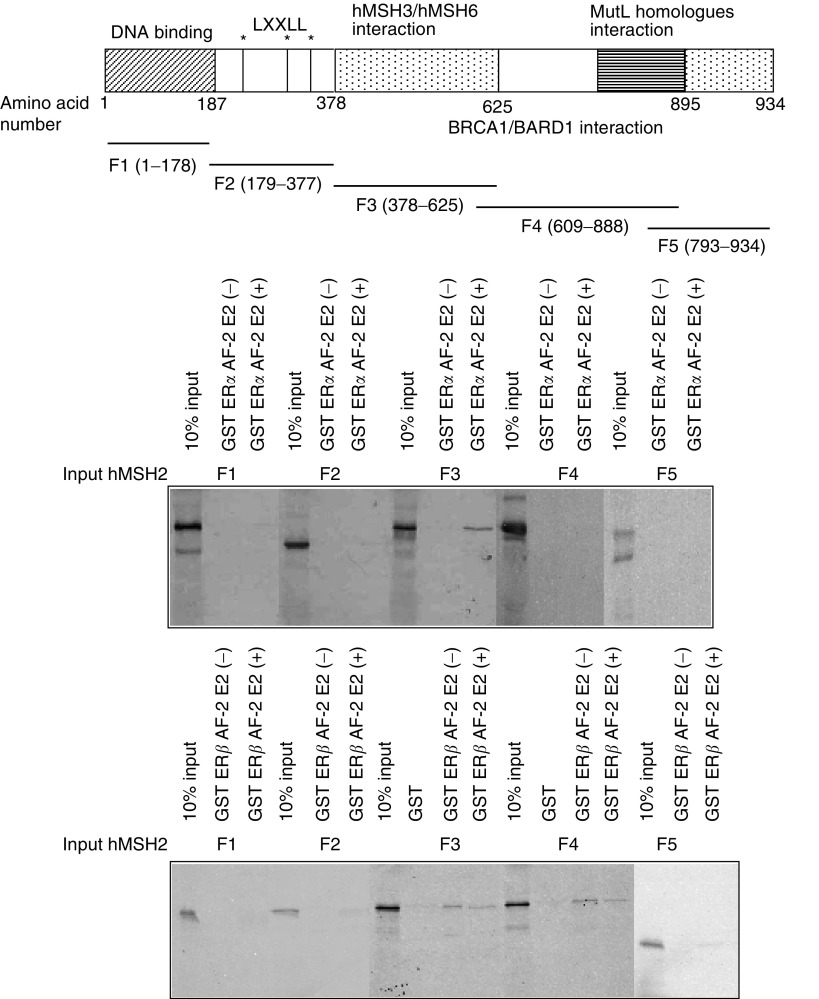
Mapping of the ER *α*/*β*-interacting region of hMSH2. A schematic diagram of the structure of hMSH2 is shown. ‘F1’, ‘F2’, ‘F3’, ‘F4’, and ‘F5’ fragments of hMSH2 were *in vitro* translated. In view of the result of Figure 1, the fragments of hMSH2 and GST-ER *α*/*β* AF-2 were tested for detection. The mixtures were washed and subjected to SDS–PAGE and then visualised by autoradiography.

To map the region of hMSH2 that interacted with ER *α*/*β* AF-2, a GST pulldown assay was performed using GST-fused ER *α*/*β* AF-2, and *in vitro* translated hMSH2 fragments (Figure 2). The FLAG-tagged hMSH2 fragments, abbreviated ‘F1’ (1–178), ‘F2’ (179–377), ‘F3’ (378–625), ‘F4’ (609–888), and ‘F5’ (793–934), were *in vitro* translated and tested for the interaction with ER *α*/*β*. Only F3 region of hMSH2 interacted with ER *α* AF-2 in a ligand-dependent manner. Thus, F3 of hMSH2 complexed with ER *α* via the hMSH3/hMSH6 interaction domain. Interestingly, the liganded ER *α* column did not retain F2, which contains LXXLL motifs thought to interact with liganded NRs (Heery *et al*, 1997; Voegel *et al*, 1998). F3 and F4 regions of hMSH2 interacted with ER *β* AF-2 in a ligand-independent manner. Thus, F3 and F4 of hMSH2 complexed with ER *β* via the hMSH3/hMSH6 interaction domain.

### hMSH2 potentiates the transactivation function of ER *α* in a ligand-dependent manner

**Figure 3:**
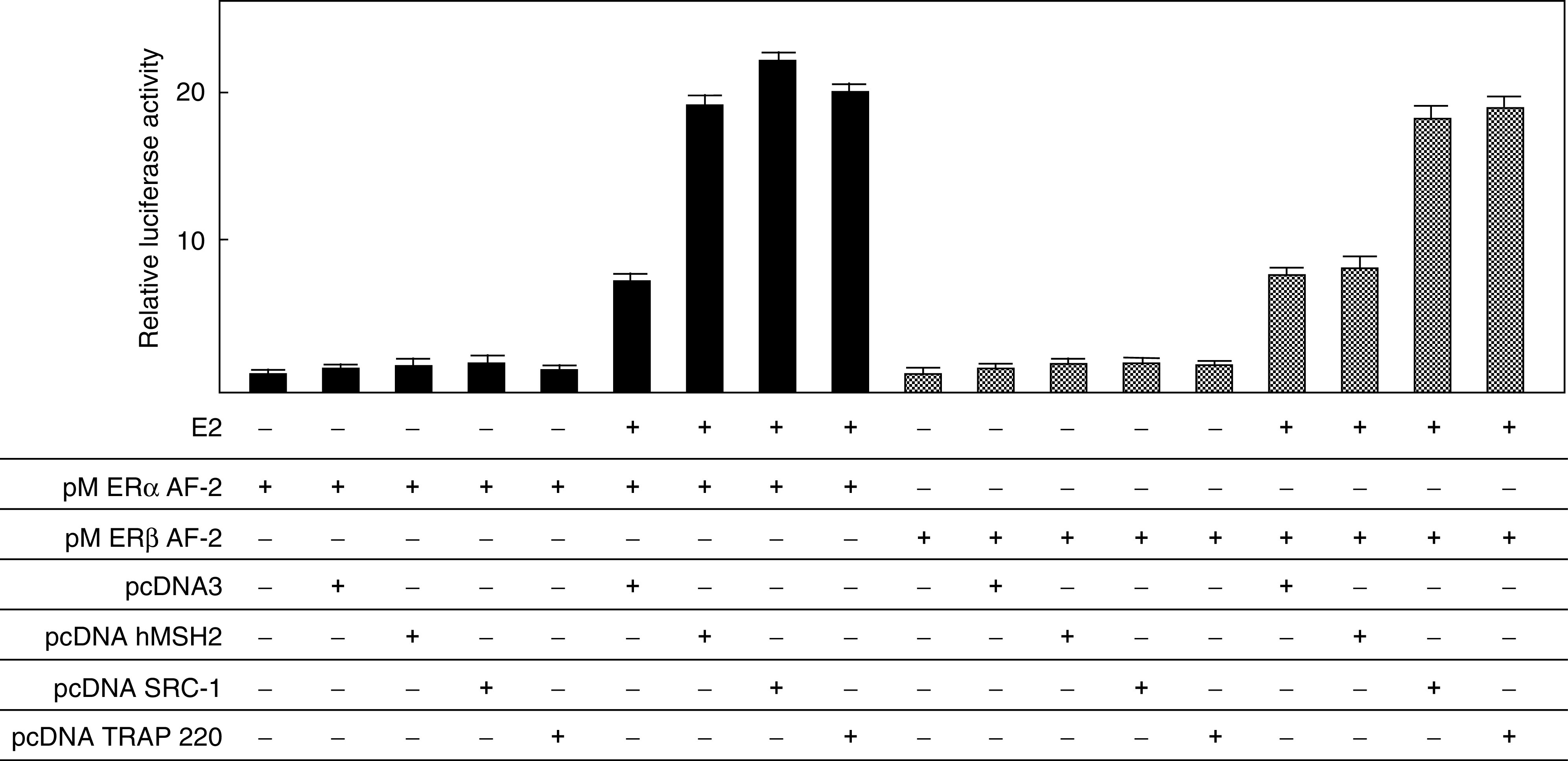
Activation of transcription by ER *α* AF-2 by the overexpression of hMSH2 in a ligand-dependent manner. HEC59 cells were transfected with pM ER *α*/*β* AF-2 (250 ng), 17M8 *β*-globin-luc (250 ng), pRL CMV-Luc (5 ng), pcDNA (100 ng), pcDNA hMSH2 (100 ng), pcDNA SRC-1 (100 ng), and pcDNA TRAP220 (100 ng) in the presence of 17-*β* estradiol at 10^−8^ M, and cell extracts were used for luciferase assay. Results are shown as the mean±s.d. SRC-1, TRAP 220, and hMSH2 caused a ligand-dependent potentiation of the ER *α* transactivation function.

To examine the coactivator activity of hMSH2 in the transactivation function of ER *α*/*β*, a transient transfection assay was performed using a luciferase reporter plasmid driven by the *β*-globin promoter containing GAL4 DNA-binding domain. While ER *α*-GAL4 fusion protein (GAL-ER *α* AF-2) showed a ligand-dependent transactivation function in human HEC59 endometrial cancer cells, a transient coexpression of hMSH2 led to an approximately 2.5-fold increase in the luciferase activity compared to that of GAL-ER *α* AF-2 alone (Figure 3). The promoted transactivation function by hMSH2 was comparable to that by AF-2 coactivators, SRC-1 and TRAP220. This enhancement of transactivation by hMSH2 was observed in hMSH2-deficient HEC59 cells, but not in the other cell lines, MDA-MB-231 and T-47D (data not shown). The ligand-induced transactivation was not promoted by hMSH2 when GAL-ER *β* AF-2 was used (Figure 3), as expected from the *in vitro* ER *α*/*β* experiments. This again suggested a significant role of hMSH2 in the ligand-dependent transactivation function of ER *α*.

## DISCUSSION

We showed that endogenous ER *α*/*β* associated with wild-type hMSH2 in the cultured human cells. Immunoblotting revealed the exsistence of hMSH2 in the cell lysate immunoprecipitates, which was confirmed by reciprocal immunoprecipitation with the specific antibody raised against hMSH2. The direct interaction between ER *α*/*β* and hMSH2 was investigated by GST pulldown assays. The GST-fused ER *α* AF-2 protein showed the ability to retain hMSH2 in a ligand-dependent manner. F3 region of hMSH2 interacted with ER *α* AF-2 in a ligand-dependent manner, which was supported by the findings that ER *α* alone potently enhanced hMSH2 transactivation function. In contrast, F3 and F4 regions of hMSH2 interacted with ER *β* AF-2 in a ligand-independent manner, and ER *β* lacked the ability to enhance hMSH2 transactivation function. These results indicated that the ER *α*/*β*-containing complexes might play a role in the MMR system of hMSH2.

Ligand-dependent interaction of hMSH2 was identified as a unique property because hMSH2 directly binds to ER *α* AF-2 as if hMSH2 acts as an ER coactivator. Although hMSH2 has three NR recognition motifs (LXXLL, NR box), these motifs have no significant role in the binding of ER *α* and ER *β*. Given that hMSH2 may promote gene regulation with ERs, and that the mutations in hMSH2 may lead to an altered ER *α*-dependent gene regulation and an altered tumorigenesis in oestrogen-dependent cancers (presumably through the modulation of ER-mediated oestrogen signalling), the interaction between ER *α*/*β* and hMSH2 may partially account for the relationship between HNPCC and oestrogen-dependent extracolonic tumours such as endometrial cancer. Our results also indicated that hMSH2 competed with ER *α* in terms of transcriptional control via the hMSH3/hMSH6 interaction domain of hMSH2, which suggestes the possibility that the common coactivator complexes of ER, including the p160 family complex, TRAP/DRIP complex and TFTC complex, might have a functional relationship with the MMR complex.

Oestrogen stimulates diverse biological effects in humans, and many of these effects result from a direct interaction of oestrogen with an NR that activates the expression of genes encoding proteins with important biological functions. Oestrogen has a mitogenic action in hormone-sensitive tissues such as the uterus. In addition to this action, oestrogen possesses carcinogenic activity. Prolonged exposure to oestrogen without opposing by progesterone is considered to cause endometrial hyperplasia and endometrioid adenocarcinoma in relatively young women. The interaction between ER *α*/*β* and hMSH2 might have a significant role in the pathogenesis of endometrial cancer. Recently, it was reported that not only tamoxifen, a selective oestrogen receptor modulator (SERM) used in the treatment of breast cancer, but also oestrogen derivatives possess a high mutagenic potential (Mizutani *et al*, 2004), which is attained by two mechanisms. First, DNA replication blocks may cause chromosomal breaks and lead to translocation or large deletion. Second, the employment of error-prone translesion DNA synthesis polymerases to bypass DNA damage is likely to result in an accumulation of point mutations. On the other hand, withdrawal of oestrogen may increase a risk of microsatellite instability-positive colon cancer based on the epidemiological data (Slattery *et al*, 2001). Considering the present data, ER itself may have a fuctional role in DNA MMR. Especially, ER *β*, which is expressed in colon cancer (Gustafsson, 2003), may have a potent role through the ligand-independent interaction with hMSH2.

The present study showed that ER *α*/*β* bound directly to wild-type hMSH2. Considering the fact that most mutations found in the patients of HNPCC result in a premature truncation of hMSH2, the loss of hMSH3/hMSH6 interaction domain in hMSH2 may result in a loss of interaction between ER *α*/*β* and hMSH2. In this respect, the functional interaction between ER*α*/*β* and hMSH2 may be required to prevent or promote the progression in the pathogenesis of endometrial cancer complicating HNPCC. Our transient transfection assay showed that the MMR complex clearly served as a coactivator complex in the promoters of ER *α* target genes because hMSH2 enhanced ER *α*-mediated ligand-dependent transactivation function. Further investigations should be performed to elucidate the molecular mechanisms that underlie the formation of ER*α*/*β*-hMSH2 complexes in normal cell growth, thereby revealing hMSH2 as a tumour suppressor in oestrogen-responsive organs, especially the endometrium. In conclusion, these results suggest that the failure of binding between hMSH2 and ER *α*/*β* may be a key event in cancer predisposition.
